# Implantation of 3D Constructs Embedded with Oral Mucosa-Derived Cells Induces Functional Recovery in Rats with Complete Spinal Cord Transection

**DOI:** 10.3389/fnins.2017.00589

**Published:** 2017-10-31

**Authors:** Javier Ganz, Erez Shor, Shaowei Guo, Anton Sheinin, Ina Arie, Izhak Michaelevski, Sandu Pitaru, Daniel Offen, Shulamit Levenberg

**Affiliations:** ^1^Department of Human Molecular Genetics and Biochemistry, Felsenstein Medical Research Center, Sackler School of Medicine, Tel Aviv University, Tel Aviv, Israel; ^2^Department of Biomedical Engineering, Technion, Haifa, Israel; ^3^Department of Neurobiology, The George S. Wise Faculty of Life Sciences, Tel Aviv University, Tel Aviv, Israel; ^4^Department of Oral Biology, School of Dental Medicine, Tel Aviv University, Tel Aviv, Israel; ^5^Department of Molecular Biology, Faculty of Natural Sciences, Ariel University, Ariel, Israel

**Keywords:** spinal cord injury, oral mucosa, stem cells, tissue engineering, regenerative medicine

## Abstract

Spinal cord injury (SCI), involving damaged axons and glial scar tissue, often culminates in irreversible impairments. Achieving substantial recovery following complete spinal cord transection remains an unmet challenge. Here, we report of implantation of an engineered 3D construct embedded with human oral mucosa stem cells (hOMSC) induced to secrete neuroprotective, immunomodulatory, and axonal elongation-associated factors, in a complete spinal cord transection rat model. Rats implanted with induced tissue engineering constructs regained fine motor control, coordination and walking pattern in sharp contrast to the untreated group that remained paralyzed (42 vs. 0%). Immunofluorescence, CLARITY, MRI, and electrophysiological assessments demonstrated a reconnection bridging the injured area, as well as presence of increased number of myelinated axons, neural precursors, and reduced glial scar tissue in recovered animals treated with the induced cell-embedded constructs. Finally, this construct is made of bio-compatible, clinically approved materials and utilizes a safe and easily extractable cell population. The results warrant further research with regards to the effectiveness of this treatment in addressing spinal cord injury.

## Introduction

Spinal cord injury (SCI) results in structural and functional damage to neural circuitry, arising from axon loss, local inflammation, glial scarring, and progressive tissue cavitation extending beyond the boundaries of the primary lesion (Cregg et al., 2014). Allograft nerve transplantation, cell therapy, and implantation of engineered tissue have achieved partial functional recovery in SCI rodents (Ramon-Cueto et al., 2000; Coumans et al., 2001; Cao et al., 2004; Fouad et al., 2005; Guo et al., 2007; Pan et al., 2008; Lu et al., 2012, 2014). Cell therapy relying on autologous cells capable of inducing neuroprotective and regenerative processes would be of significant potential for future clinical purposes. Several types of autologous cells present attributes rendering them promising candidates for effective cell therapy. Mesenchymal stromal cells from various sources and olfactory ensheathing cells are the most studied cell types for autologous transplantation, showing efficacy to ameliorate SCI in animal models and also in early human clinical trials (Tabakow et al., 2013; Jarocha et al., 2014; Kakabadze et al., 2016; Assinck et al., 2017; Melo et al., 2017). These can be used in combination with scaffolds, genetic engineering, medium-based induction or co-transplanted with other cell types (Assinck et al., 2017). Other cell types such as induced pluripotent-derived cells (Khazaei et al., 2016), dental pulp stem cells (Sakai et al., 2012), neural progenitors grafts (Lu et al., 2014; Kadoya et al., 2016) showed efficacy in treating SCI in animal models. Human oral mucosa stem cells (hOMSCs) exhibit a neural crest-like stem cell phenotype, high expandability, which persists for over 70 cumulative population doublings, low interdonor heterogeneity and a negligible effect of aging on clonogenicity, growth and differentiation (Marynka-Kalmani et al., 2010). We recently reported that hOMSCs can be induced into astrocyte-like cells, which exhibit elevated secretion of neurotophic factors (NTFs), provide neuroprotection to motor neurons *in vitro* and enhance neural repair in rats with sciatic nerve injury (Ganz et al., 2014).

Tissue engineered (TE) scaffolds provide a 3D environment in which cells can attach, grow and differentiate, maintain cell distribution, and provide graft protection following transplantation (Levenberg and Langer, 2004). Biodegradable scaffolds are of special importance for spinal cord repair since they can provide the initial protection for grafted cells and guidance for axons, while degrading after these processes are completed. We have shown that biodegradable poly(l-lactic acid)(PLLA)/polylactic-glycolic acid (PLGA) scaffolds support proliferation, differentiation, and organization of embedded olfactory bulb-derived cells, enhancing their NTF secretion (Blumenthal et al., 2013; Shandalov et al., 2014). PLGA was selected to provide flexibility, whereas the PLLA was chosen to provide stiffness. Thus, the biomechanical properties of the scaffold depend on the ratio of PLLA and PLGA used, allowing tuning of its stiffness and its micro-pores' shape (Levy-Mishali et al., 2009; Lesman et al., 2011). For this study we used 50% PLLA and 50% PLGA porous scaffolds, which feature porous structure compatible with cell culture, easily implantable, and estimated to degrade in ~60 days (Teng et al., 2002).

Furthermore, such scaffolds can act as a reservoir for secreted NTFs, creating gradients capable of both supporting morphogenesis and potentiating their actions (Blumenthal et al., 2013). Based on this evidence, we hypothesized that an implantable TE construct consisting of induced-hOMSCs embedded in a fibrin/PLLA/PLGA matrix may act as a multi-effector device capable of enhancing endogenous regenerative processes and promoting neurological recovery following complete spinal cord transection.

## Materials and methods

### Naïve hOMSC cell culture

After obtaining signed informed consent and the approval of the Institutional Helsinki Committee at the Baruch Padeh Medical Center, Poria, Israel, hOMSCs were isolated from oral mucosa biopsies, collected by Dr. Shareef Araidy and Dr. Sammy Pour. Briefly, biopsies were incubated overnight at 4°C in dispase (Sigma, Israel). Then, the epithelial layer was separated from the lamina propria and the latter was minced into 0.5 mm^3^ pieces and placed in 35 mm culture dishes (Nunc). Expansion medium was gently added to the explants to enhance their attachment to the floor of the dish. Cells that migrated from the explant to the culture dishes were harvested with 0.25% trypsin (Biological Industries, Beit-Haemek, Israel) and seeded at a cell density of 4 × 10^4^ cells/cm^2^. Cells were passaged at 70–80% confluence. hOMSCs were cultured in expansion medium consisting of low-glucose Dulbecco's modified Eagle's medium, supplemented with 100 μg/ml streptomycin, 100 U/ml penicillin (Biological Industries, Beit-Haemek, Israel), 2 mM glutamine (Invitrogen, Carlsbad, CA, USA) and 10% fetal calf serum (FCS) (Gibco), as described by Marynka-Kalmani et al. (2010). All experiments used hOMSCs at passages 4–10. Three different donor tissues were used to form the constructs tested.

### PLLA/PLGA scaffold preparation

Porous sponges composed of 50% PLLA and 50% PLGA were fabricated utilizing a particulate leaching technique to achieve pore sizes of 212–600 μm and 93% porosity. Briefly, PLLA (Polysciences) and PLGA (Boehringer Ingelheim) were dissolved 1:1 in chloroform to yield a 5% (w/v) polymer solution; 0.24 ml of this solution was loaded into molds packed with 0.4 g sodium chloride particles. The solvent was allowed to evaporate overnight, and the sponges were subsequently immersed, for 8 h, in distilled water (changed every hour) to leach the salt and create an interconnected, porous structure. Final PLLA/PLGA sponges were 3 × 3 mm, and 1 mm thick. Before use, sponges were soaked overnight in 70% (v/v) ethyl alcohol and then three times in PBS. Previous works have demonstrated the biocompatibility and biodegradability of PLLA/PLGA porous scaffolds and estimated its degradation time to be about 30–60 days (Teng et al., 2002).

### hOMSC seeding and differentiation

**Figure 1:**
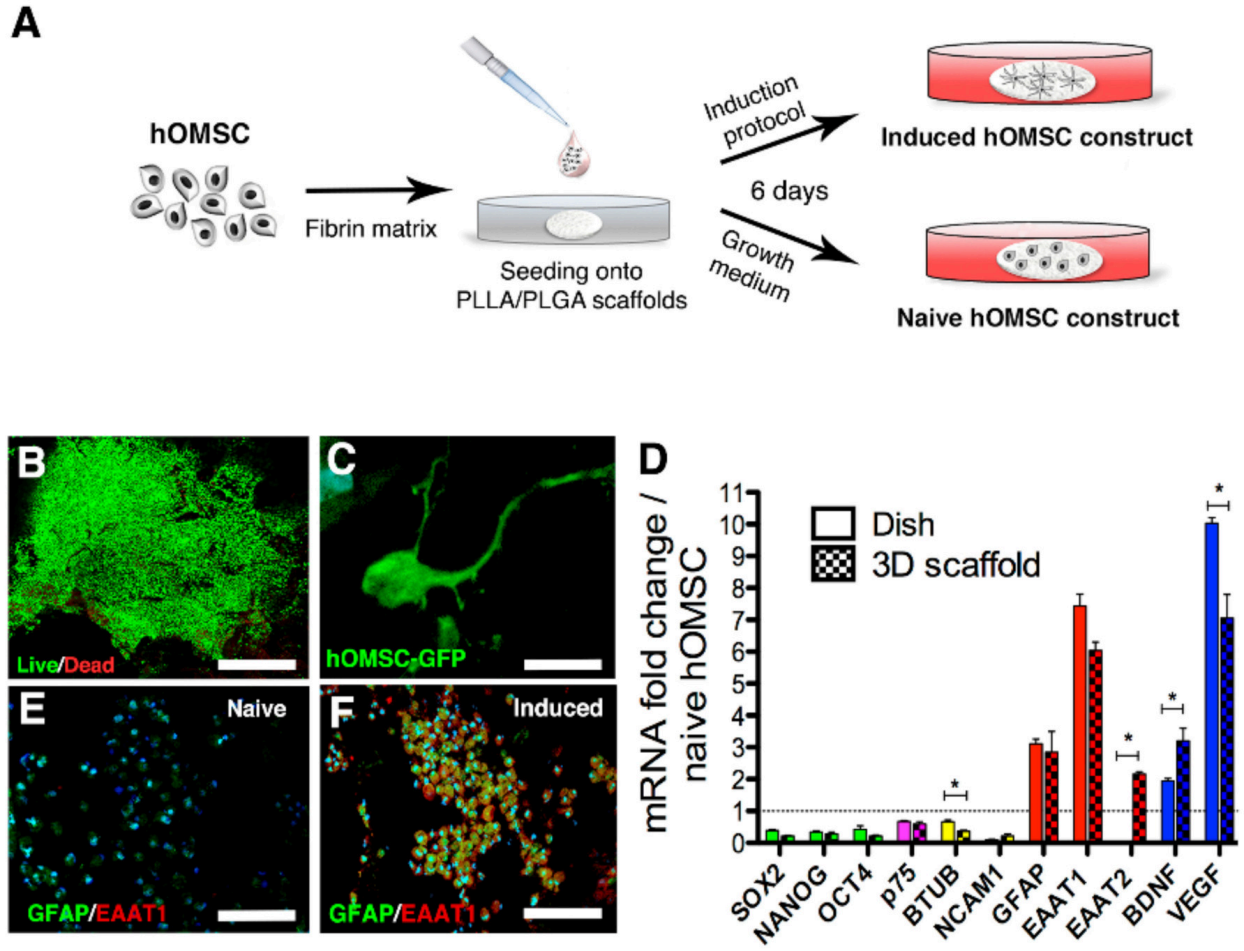
Characterization of hOMSC constructs. **(A)** Preparation scheme of naïve and induced hOMSC constructs. **(B)** Viability of hOMSC after on-scaffold induction (green indicates viable cells and red indicates dead cells), scale bar = 500 μm. **(C)** GFP signals showing cellular projections in hOMSC-GFP engineered cells after induction, scale bar = 10 μm. **(D)** RT-PCR-based comparison between 3D on-scaffold-induced hOMSCs (dotted bars) and hOMSCs induced in culture plates (solid bars): pluripotency and neural crest markers (green and magenta), neuronal markers (yellow), astrocytic markers (red), and neurotrophic factors (blue). Bars represent fold-increase compared to naïve hOMSCs (mean ± SEM). Statistical differences between 2D and 3D induced hOMSC (*n* = 3/group) were assessed by *T-*test (^*^p < 0.05). Astrocyte markers GFAP (green) and EAAT1 (red) in naïve hOMSC constructs **(E)** and induced constructs **(F)** (scale bar = 100 μm, *n* = 5/group).

Naïve hOMSCs were harvested with trypsin (Biological Industries, Israel), counted and aliquoted (5 × 10^5^ cells/tube). Cells were suspended in 3.5 μl human thrombin (Sigma, Israel), followed by addition of 3.5 μl human fibrinogen (Sigma, Israel), and then immediately seeded on PLLA/PLGA scaffolds in a non-tissue culture plate. Acellular scaffold were seeded with fibrinogen and thrombin without cells. We allowed cells in seeded scaffolds to attach and fibrin to polymerize for 30 min in 37°C, 5% CO_2_, at high humidity. hOMSC expansion medium (1 mL) was then added to each scaffold. The next day, a two-step medium-based consisting the first of basic fibroblast growth factor 2 (R&D Systems) and 20 ng/ml epidermal growth factor (R&D Systems) was started. Following 72 h, the second differentiation step was initiated. Cells were incubated in serum-free medium (DMEM low glucose/SPN/glutamine) with the addition of 1 mM dibutyryl cyclic AMP (Sigma-Aldrich), 0.5 mM 3-isobutyl-1-methylxanthine (IBMX) (Sigma-Aldrich), 50 ng/ml Neuregulin, and 1 ng/ml platelet-derived growth factor (PDGF; PeproTech Asia, Rehovot, Israel) for an additional 72 h. Acellular scaffolds were cultured for 6 days in growth medium (Figure 1A).

### Viability assay

To assess cell viability, scaffolds were loaded with calcein acetoxymethyl ester (calcein AM; 1 μmol/L) and ethidium homodimer-1 (4 μmol/L) (Sigma-Aldrich) for 50 min at 37°C, on a 3D XYZ shaker. Scaffolds were then washed thrice with PBS and visualized using a confocal microscope. Cell viability was assessed before implantation.

### Real-time reverse transcription PCR

Total RNA from hOMSCs induced on scaffolds or in cell culture plates (*n* = 3/group) was isolated using the TRI reagent (Invitrogen, Carlsbad, CA, USA), according to the supplier's recommendations. RNA (2 μg) was reverse transcribed with random primers and SuperScriptIII (Invitrogen, Carlsbad, CA, USA). Real-time PCR of the genes of interest was performed in a StepOnePlus^TM^ (Applied Biosystems), using PlatinumR SYBRR Green qPCR SuperMix UDG with ROX (Invitrogen, Carlsbad, CA, USA). PCR amplification was performed over 40 cycles (program: 2 min at 50°C, 2 min at 95°C, 40 repeats of 15 s at 95°C and 30 s at 60°C). Data were quantified using the ΔΔCt method, and normalized to the lactate dehydrogenase A (LDHA) housekeeping gene. ΔCt of naïve cultures served as baseline values. Data are presented as the mean ± standard error of the mean (SEM) change from the baseline.

### Cytokine array

Cytokine levels in conditioned medium of naïve and induced constructs were compared using the human RayBio® G-Series Cytokine Array (RayBiotech, Inc., USA), as per the manufacturer's guidelines. Total cell protein served as the normalization factor between conditions. Naïve hOMSCs served as the reference and results are expressed as fold-change from naïve conditions per milligram of protein.

### Spinal cord injury and construct implantation

**Figure 2:**
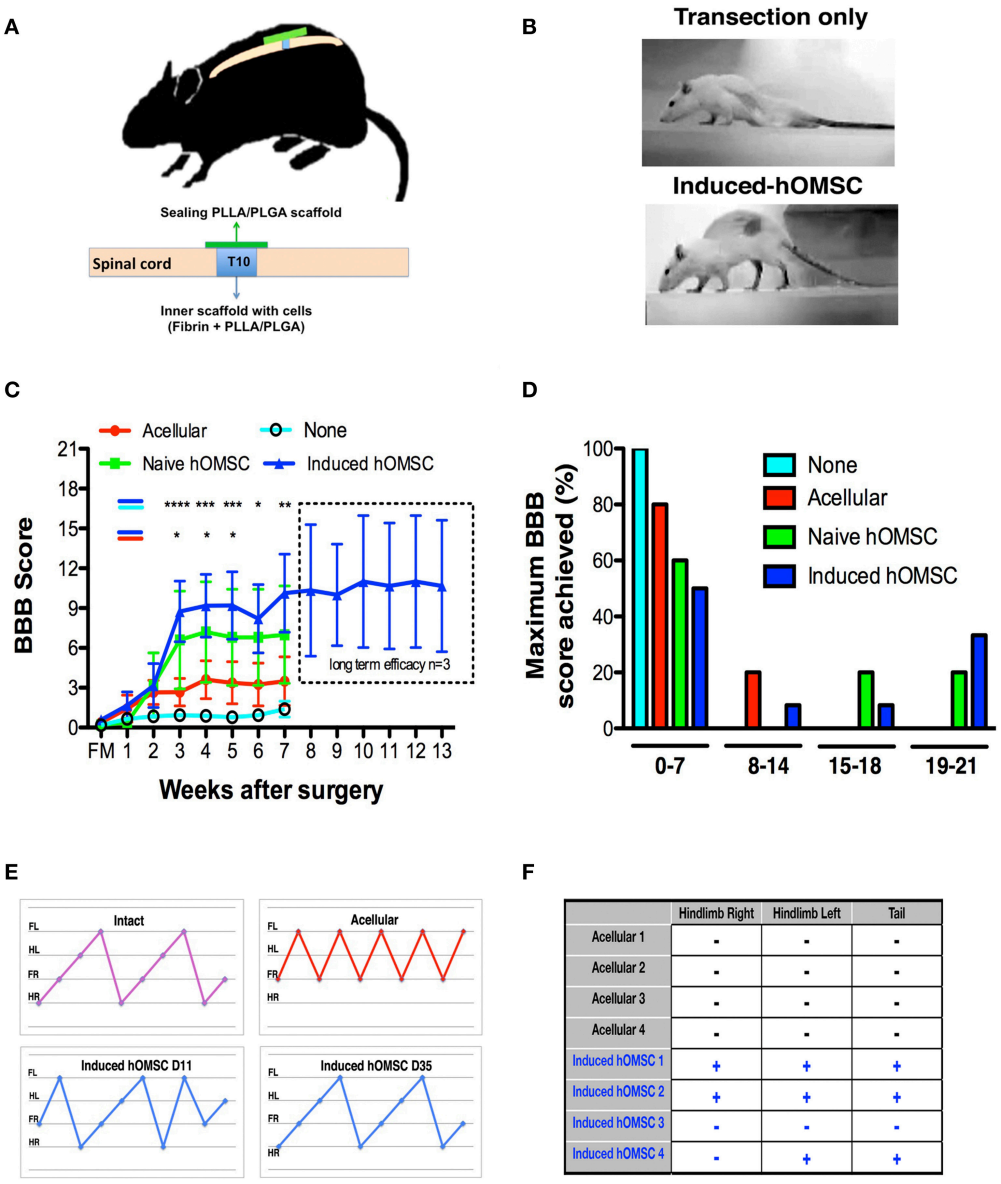
*In-vivo* analysis of therapeutic effects of implanted induced constructs. **(A)** Implantation scheme. Following complete transection at T10, cell-embedded, or acellular scaffolds are implanted in the transection site and sealed with an acellular PLLA/PLGA scaffold. **(B)** Representative images of rat posture 43-days following implantation of an induced-construct (bottom) vs. transection only (top). **(C)** BBB scores over time of rats treated with induced constructs (*n* = 12, blue), naïve constructs (*n* = 5, green), acellular scaffolds (*n* = 10, red), or left untreated (none, *n* = 16, cyan). The dotted line indicates the results of a long-term efficacy study for the last group treated with induced constructs (*n* = 3). Data are presented as mean ± SEM, and two-way ANOVA with Bonferroni *post-hoc* test evaluated statistical differences between the four groups over time (^*^p < 0.05, ^**^p ≤ 0.01, ^***^p < 0.001, ^****^p ≤ 0,0001 between induced and acellular constructs: blue-red indication; between induced constructs and none: blue-cyan indication). **(D)** Maximum BBB score per experimental group histogram **(E)** Coordinated gait analysis of intact (upper left), acellular (upper right), and induced construct groups, on days 11 (lower left) and 35 (lower right) post-implantation. Gait pattern legend- hind-right (HR), front right (FR), hind left (HL), front left (FL). **(F)** Results of the nociceptive perception test of the hind limbs and tail in rats treated with induced constructs vs. acellular scaffolds (+: responsive, −: unresponsive to the stimulus, *n* = 4/group).

All animal experiments were performed in strict compliance with protocols approved by the Technion—Israel Institute of Technology and Tel Aviv University Ethics Committees (IL 040032012). In line with these guidelines, rats that performed self-mutilation or lost more than 20% body weight after surgery were excluded from the experiment. Animal randomization was performed by an experimenter blinded to the treatments. Adult female Sprague-Dawley rats were anesthetized with a mixture of xylazine (10–15 mg/kg) and ketamine (60–90 mg/kg) and with a maintenance dose of isoflurane (Harvard Apparatus, USA) during surgery. After laminectomy at the 9th−11th thoracic vertebral levels, the spinal cord was completely transected at the T10 level, using a microscissor (Kent Scientific, USA). The rostral and caudal stumps were lifted to ensure complete transection and a hook (Kent Scientific, USA) was passed circularly inside the generated gap to confirm that no fibers remained at the bottom part of the spine canal. The groups evaluated in this study were designated as none (no scaffold or cells), acellular scaffold (scaffold with no cells), naïve (scaffold with naïve hOMSC), and induced (scaffold with induced hOMSC into a neural phenotype). The lesion in rats assigned to the “none” group was then sutured as described below. For the other treatment groups, PLLA/PLGA scaffolds were inserted between both caudal and rostral parts of the spinal cord and sealed with an acellular PLLA/PLGA scaffold. An additional PLLA/PLGA scaffold was placed over the transected site and over the exposed rostral and caudal regions of the cord to provide structural support and minimize friction between the spinal cord and the vertebra bone (Figure 2A). Muscle layers and skin were then sutured and the rats were placed in temperature-controlled incubation chambers until they awoke. They were then transferred to cages, and bladder massages were applied twice daily until bladder function was regained. Antibiotics (Solvasol, 10 mg/kg body weight) were injected daily for 1 week. Buprenorphine (Bayer) was administered at a dose of 0.01–0.05 mg/kg before surgery and 3 days after. Ciclosporin (10 mg/kg/d) (Novartis) was administered daily to all rats 1 day before surgery and for 5 days post-surgery. The identity of each rat within each experimental group remained coded until the end of the experiment and data analysis. Surgeries were carried out by three independent investigators and crosschecked to minimize influence of human factors.

### Motor analysis in spinal cord injury

Two independent blinded observers, who did not participate in the surgeries or construct implantation, evaluated and scored rat behavior. Rats were given random numbers and the examiners were blinded to treatments, reporting results per rat without any further information regarding the treatment each animal received. Functional recovery and gait was assessed and scored using BBB tests (Basso, Beattie, and Bresnahan locomotor scale method; de Medinaceli et al., 1982; Basso et al., 1995). Throughout the experiment, the following exclusion criteria were applied: (a) A BBB ≥4 three days after surgery, indicated partial transection. (b) Self-mutilation resulting in loss of one digit or more of the hind limbs. (c) Substantial weight loss (see Supplementary Table 2). The assays were performed on a setup enabling simultaneous photography of the sagittal and coronal planes. Measurements were made 1–4 d following implantation, followed by measurements every 7 days. All measurements were made at the same time of day to avoid circadian variability. Baseline BBB was defined as the value recorded at the first test after surgery. The weekly score was the lowest score obtained during each calendar week.

### Immunofluorescence

#### Naïve and induced hOMSC analysis

Cells were fixed in 4% PFA (*n* = 5 scaffolds/group). Samples were blocked with 5% goat serum, 1% BSA, and 0.05% Triton-X in PBS for 2 h, and then incubated with rat anti-GFAP (1:5,000, Life sciences, 13-0300, clone 2.2B10, Zoltewicz et al., 2012) and rabbit anti-EAAT1 (1:1,000, Abcam, ab416, Gunn et al., 2013), overnight at 4°C. Then, samples were incubated with dye-conjugated secondary antibodies (Alexa 488 and 647), goat anti-rat (H+L) (Molecular probes, A-11006) and goat anti-rabbit (H+L) (1:1,000, Molecular probes, A-11036). Nuclear DNA was stained with 4,6-diamino-2-phenylindole (DAPI) (1:1,000, SIGMA).

#### Spinal cord analysis

Rats were sacrificed with CO_2_ and immediately perfused with 4% PFA. Spinal cords were dissected, embedded in optimal cutting temperature compound (OCT) and longitudinally sectioned (20 μm) using a cryostat (Leica CM1850, Germany). Five interspaced sections from five animals in each respective group, were used for the analysis. Sections were blocked with 5% goat serum, 1% BSA, and 0.05% Triton-X in PBS for 2 h, and then incubated with primary antibodies overnight at 4°C. The following primary antibodies were used: rabbit anti-b III-tubulin (1:2,000, Abcam, ab18207, Chen et al., 2010), rabbit anti-GAP-43 (1:1,000, Millipore, Kawaja et al., 2011), rat anti-GFAP (1:5,000, Life sciences, 13-0300, clone 2.2B10, Zoltewicz et al., 2012), rat anti-CD11b (1:1,000, Abcam, ab8878, clone M1/70, Fulmer et al., 2014), rat anti-MBP (1:1,000, Abcam, ab62631, clone MBP101, Kwon et al., 2014), mouse anti-human nuclei (1:2,000, Millipore, MAB1281, clone 235-1, Varela et al., 2012), mouse anti-CSPG (1:500, Millipore, MAB5284, clone cat-301, Andrews et al., 2012), mouse anti-NF200 (1:1,000, Abcam, ab24574, Tokuda et al., 2015) and mouse anti-nestin (1:200, Millipore, MAB353, clone rat-401, Lee et al., 2012). For immunofluorescence staining, sections were incubated with Alexa 488-conjugated goat anti-mouse (H+L) (1:500, Molecular probes, A-11001), goat anti-rabbit (H+L) (1:500, Molecular probes, A-11034) or goatanti-rat (H+L) (1:500, Molecular probes, A-11006) antibodies, or with Alexa 568-conjugated goat anti-mouse (H+L) (1:500, Molecular probes, A-11031) or goat anti-rabbit (1:500, Molecular probes, A-11036). Nuclear DNA was stained with DAPI (1:1000). For immunohistochemistry, hematoxylin, and eosin staining was performed. Sections from the same rats were used for immunochemistry and immunofluorescence analyses. Using custom-made MATLAB software, the region of interest (ROI) was manually defined to exclude the non-scaffold area. The resultant image was decomposed to blue, green, and red channels. For each channel, an adaptive threshold filter was applied at 35% of the maximum intensity value to remove noise. Subsequent processing and noise filtration were achieved by applying the erosion morphological operator. To quantify the relative area covered by an IHC indicator, the total pixelated area was calculated and normalized to the actual area of the ROI. To compare the myelination of axons between the groups we evaluated and quantified expression of MBP in elongated areas, suggesting presence of myelinated axons. MATLAB scripts were programmed to automatically count elongated elements representing axons in MBP immunofluorescence images. Images were cleaned using morphological operators (erosion). The resultant binary image was segmented by selecting connected areas. Areas larger than a certain threshold were automatically excluded from the ROI to avoid miscalculation of large bundles of connected neurons. For each region, second-order moments were calculated to obtain major and minor axis lengths. All areas containing a major to minor axis ratio >5 indicating the matching ellipse has one axis at least five times larger than the other axis were identified as elongated axons. The number of elongated axons in each image was counted. Data are presented as mean ± SEM, One-way ANOVA, with the Bonferroni *post-hoc* test, evaluated statistical differences between the groups (^*^p < 0.05, ^**^p < 0.01, ^***^p < 0.001).

### MRI-DTI

#### MRI protocol

Magnetic resonance imaging (MRI) was performed, with the assistance of Bioimage Ltd., in a 7T MRI system (Bruker, Germany), using a 20 mm surface coil placed on the back of the rat, at the injury site. Rats were anesthetized using 1–3% isoflurane and maintained at 37°C; breathing was monitored with a respiratory sensor (*n* = 3/group). The MRI protocol included the following sequences:

#### T2 RARE

Sagittal T2-weighted imaging was performed in order to localize the axial slices in the correct location, including upstream and downstream regions adjacent to the injury site. T2 RARE included the following parameters: TR/TE = 1,200/16, RARE factor = 4, no. of averages = 4, 20 slices of 0.8 mm, in-plane resolution of 0.17 × 0.2 mm (matrix size 128 × 128 and FOV of 25.6 × 22.8 mm).

#### DTI

DTI was performed under the following conditions: TR/TE = 4,500/30 ms, 4 EPI segments, Δ/δ = 10/4.5 ms, 15 non-collinear gradient directions with a single b value shell at 1,000 s/mm^2^ and one image with a b value of 0 s/mm^2^ (referred to as b0), 3 averages, 2 repetitions. Geometrical parameters were: 18 slices of 1 mm thickness (brain volume) and in-plane resolution of 0.156 × 0.156 mm^2^ (matrix size of 128 × 128 and FOV of 20 mm^2^). The duration of each DTI repetition was 14:24 min.

#### DTI fiber tracking

DTI calculation and fiber tracking were performed using the ExploreDTI software (Leemans et al., 2009). The tensors obtained were spectrally decomposed to their eigen-components. The eigen-values were used to calculate FA maps. Tractography was applied using Deterministic (streamline) fiber tracking, terminating at voxels with an FA lower than 0.3 or following a tract orientation change higher than 30° (Basser et al., 2000). Fibers that passed through a manually selected ROI were plotted. The fibers were plotted as streamlines. The masks obtained were overlaid over the color-coded FA image.

### CLARITY and lightsheet imaging

The CLARITY protocol was used to map the neural fiber projections in the entire spinal cord tissue (Chung et al., 2013). Firstly, the hydrogel monomer solution was prepared, on ice, by adding in deionized water with 4% acrylamide solution (Bio-Rad), 0.025% Bis-acrylamide solution (Bio-Rad), 0.25% photoinitiator VA044 (Wako), 10% 1XPBS (Gibco), and 4% PFA solution (Electron Microscopy Sciences). Then, the rats were anesthetized with ketamine and xylazine, transcardially perfused, and the spinal cords were harvested and placed in 20 ml cold hydrogel monomer solution in a 50 ml conical tube, for 7 days in a 4°C refrigerator. Next, the tubes were de-gassed in a desiccation chamber in a chemical hood to replace all of the gas in the tube with nitrogen. Later, the tubes were submerged in 37°C water bath for 3 h, to allow for hydrogel polymerization. The embedded samples were extracted from the gel in the chemical hood. To dialyze out excess PFA, initiator, and monomer, the samples were washed for 24 h at room temperature with 50 ml clearing solution (deionized water with 200 mM Boric acid (Sigma), 4% sodium dodecyl sulfate (Sigma), and NaOH pellets (EMD), pH 8.5). The samples were washed two more times with 50 ml clearing solution, for 24 h each time, at 37°C, to further reduce residual PFA, initiator and monomer. The samples were placed in clearing solution, and incubated in a 37°C water bath for 10 days until clearing cues appeared, followed by two PBST (0.1% TritonX in 1X PBS) washes, for 24 h each, in a 4°C refrigerator. The samples were then incubated at 4°C for 1 week, with primary antibodies [mouse anti-β-III tubulin (Promega, 1:2000), rabbit anti-growth-associated protein 43 (Millipore, 1:1,000)] in 5% donkey serum and 0.1% TritonX solution. After 4 consecutive 1X PBS washes, the samples were incubated at 4°C for 1 week with secondary antibodies [goat anti-mouse Cy3 (Jackson, 1:1,000), donkey anti-rabbit Alexa488 (ThermoFisher, 1:2,000)]. Then, the samples were maintained at room temperature for 2 days, in scale solution containing 4M urea, 10% glycerol, and 0.1% TX-100. Finally, the tissues were imaged using light-sheet microscopy (Zeiss Z1).

### Electrophysiology

Following ketamine/xylazine anesthesia, rats were placed in a stereotaxic apparatus and a midline incision was made in the head skin (*n* = 3/group). The cranium was exposed and two electrical stimulation screw electrodes were implanted 2 mm to the right of the midline, at −1.0 mm and +4.0 mm anterior and posterior to the bregma, respectively. The screw electrodes were connected to the output terminals of the SD9 stimulator (Grass Technologies, Warwick, RI). The sciatic nerve at the rear of the left leg was exposed and two silver wire hook electrodes were inserted. Another wire was inserted into the footpad of the leg and served as a ground electrode. The hook electrodes were connected to the unity gain headstage, built on a dual TL072 operational amplifier (Texas Instruments) and powered by two 9 V batteries. The amplified signals were band-pass filtered between 0.1 and 3 kHz (7P511 AC wideband preamplifier with 7DA driver amplifier, Grass Technologies, Warwick, RI), digitized (NI USB-6341 analog-to-digital converter, National Instruments), acquired at 10 kHz and stored on a personal computer running the WinWCP software package (courtesy of Dr. John Dempster, University of Strathclyde, UK). The stimulation intensity was chosen according to hindlimb contraction and appearance of the reliable sciatic nerve compound action potential (CAP) in the first animal, and maintained throughout the experiment.

### Sensory examination

Sensory evaluation was performed at the end of the experiment (56 days after surgery), using the pinch technique (*n* = 4/group; Gale et al., 1985). The nociceptive stimulus was applied in both hindlimbs and tail. Responses were considered binary (responsive or unresponsive, and scored as positive or negative, respectively). The responsiveness criterion was defined as a response unlikely to be purely reflexive, indicating percept of the sensory stimulation, manifested by a vocal response, head turn or a withdrawal effect of the evaluated hindlimbs or tail.

### Statistical analysis

Results were expressed as mean ± SEM. All analyses were performed using MATLAB/ Prism. Graphs were generated by Prism 5 software (USA). Differences between two groups were statistically analyzed by a two sided *T-*test assuming different variance between groups, while one-way ANOVA was applied to compare between three groups and Bonferroni comparison *post-hoc* test was used to characterize specific differences between groups. For the *in vivo* cell transplantation experiment, two-way ANOVA with Tukey's multiple comparison *post-hoc* test was performed. Significance levels: ^*^p < 0.05, ^**^p < 0.01, ^***^p < 0.001, ^****^p < 0.0001.

## Results

### Induced hoMSC-embedded constructs express and secrete neuroprotective, regenerative, and immunomodulatory factors

We first analyzed whether 3D cultures support the induction of trophic factors and astrocyte-like phenotype by mixing hOMSCs with fibrin, seeding those on PLLA/PLGA scaffolds (Supplementary Figure 1) and culturing the constructs under our previously reported astrocyte induction method originally designed for monolayer induction (Ganz et al., 2014) (Figure 1A). Our induction protocol is aimed at inducing an astrocyte-like phenotype with enhanced secretion of neurotrophic factors to act as support cells for enhancing endogenous regeneration. To assess this astrocyte-like phenotype we screened for prototypical astrocyte markers such as GFAP, EAAT1/2. Following induction (induced hOMSC), more than 95% of the cells were viable (Figure 1B) and featured elongated processes homogenously distributed within the scaffold (Figure 1C, Supplementary Video 1). Similar cellular phenotypes, characterized by decreased expression of pluripotency, neural crest and neuronal-associated genes and a substantial increase in expression of astrocyte-related genes and NTF, were observed between the hOMSCs induced in a dish vs. 3D environment. In contrast, BDNF and EAAT2 expression was significantly higher in the 3D construct, as compared to 2D cultures, while VEGF was also overexpressed, but to a lesser extent than measured in 2D-induced hOMSCs (Figure 1D). Immunofluorescence analysis of GFAP and EAAT1 staining confirmed the astrocyte-like phenotype (Figures 1E,F).

To assess the paracrinic potential of naïve vs. induced hOMSC grown on 3D constructs, we characterized the profile of secreted factors. Secreted forms of HGF, GDNF, BDNF, NT-3, VEGF, IGF-1/IGFBP3, ENA-78, and SCF-1, known to induce neuroprotection and/or regeneration, were significantly upregulated in induced vs. naïve cell-embedded constructs. In addition, several secreted factors which bear both immunomodulatory and trophic/neuroprotective/regenerative properties, such as GM-CSF, LIF, SDF-1, IL-10, IL-6, and IL-4, were also increased after induction (Supplementary Table 1).

### Implantation of induced constructs following complete spinal cord transection elicits functional recovery

The complete spinal cord transection model is characterized by severe axonal damage, associated with motor and sensory function loss caudal to the injury, and by limited spontaneous recovery (Talac et al., 2004). Immediately following complete transection of spinal cords at T10 rats were divided into the following groups: none (no scaffold or cells), acellular scaffold (scaffold with no cells), naïve (scaffold with naïve hOMSC) and induced (scaffold with induced hOMSC into a neural phenotype; Figure 2A, Supplementary Figure 2). Complete transection was confirmed with CLARITY and following IHC staining the scaffold was identified in bright field view and axons were imaged in a 3D stack using light sheet microscopy (Supplementary Figure 3).

Rats treated with constructs bearing induced hOMSCs demonstrated higher motor and sensory recovery when compared to all other groups (see Supplementary Table 2 for summary of animals participating in the study); 42% of the rats demonstrated consistent weight support of the hind limbs and statistically significant improvements in walking abilities 3 weeks after transplantation (Figures 2B–D, Supplementary Video 2–4). A two way analysis of variance was conducted on the influence of the treatment type (no construct, acellular constructs, naïve constructs and induced constructs) and time on the BBB scores. The effect of treatment was significant, yielding *F*_(3, 263)_ = 18.98 with *p* < 0.0001. The effect of time was also significant, yielding *F*_(7, 263)_ = 4.66 with *p* < 0.0001. We preformed *post hoc* analysis with Tukey multiple comparison test to assess the treatment efficacy over time. Statistically significant differences between the induced vs. the no construct and the induced vs. the acellular constructs were observed from week 3 (Figure 2C). While the improvement reached a plateau after 5 weeks, the improvement was long lasting and presented throughout 13 weeks until the experiment was terminated (Figure 2C, dashed rectangle). Brasnhan–Beatie–Basso (BBB) scores in 5/12 rats (42%) exceeded 15 points, while several rats' scores (4/12, 33%) were as high as 19–20 within the induced-construct group. In sharp contrast, none of the other treated animal groups achieved similar BBB values and untreated rats remained paralyzed throughout the experimental period. Constructs bearing naïve hOMSCs (non-induced) elicited intermediate improvements, with 1/5 rats (20%) reaching a late phase of recovery (BBB scores ≥ 17). To a much lesser extent than both cell-bearing constructs, acellular scaffolds also prompted a small improvement (1/10 rats [10%] BBB ≥14 and 8/10, 80% of rats reached BBB scores ≤7; Figure 2D). The improvement demonstrated by the induced hOMSC group was reproducible with different donors. For two additional donors induced construct yielded BBB scores >9 in 22% of the cases (2/9 rats). Furthermore, gait and coordination analysis of the recovered induced-construct-treated rats (BBB >17) revealed walking patterns similar to those of intact rats, with progressive and adaptive motor coordination over time where hindlimb—forelimb movement is followed by a hindlimb—forelimb movement from the contralateral side. This behavior developed over time in the induced group, where uncoordinated gait was present at earlier stages of recovery. In contrast, paralyzed controls exhibited movement of the forelimbs only (Figure 2E). Additionally, some of the recovered animals also exhibited a “mosey walk” or abnormal stepping pattern but yet coordinated and functional (high BBB). Coordinated gait was observed in 5 out of 12 rats treated with induced constructs, 1 out of 5 rats treated by naïve constructs, and only 1 out of 10 rats from the acellular construct group. None of the rats transected and left untreated (none group) showed any motor improvement. In addition, 56 days post-implantation, 75% of the evaluated rats treated with induced-constructs responded to gross stimuli, while rats receiving the acellular constructs failed to show any sensory response (Figure 2F).

### Functional recovery is associated with structural organization of and electric conduction through the transected site

**Figure 3:**
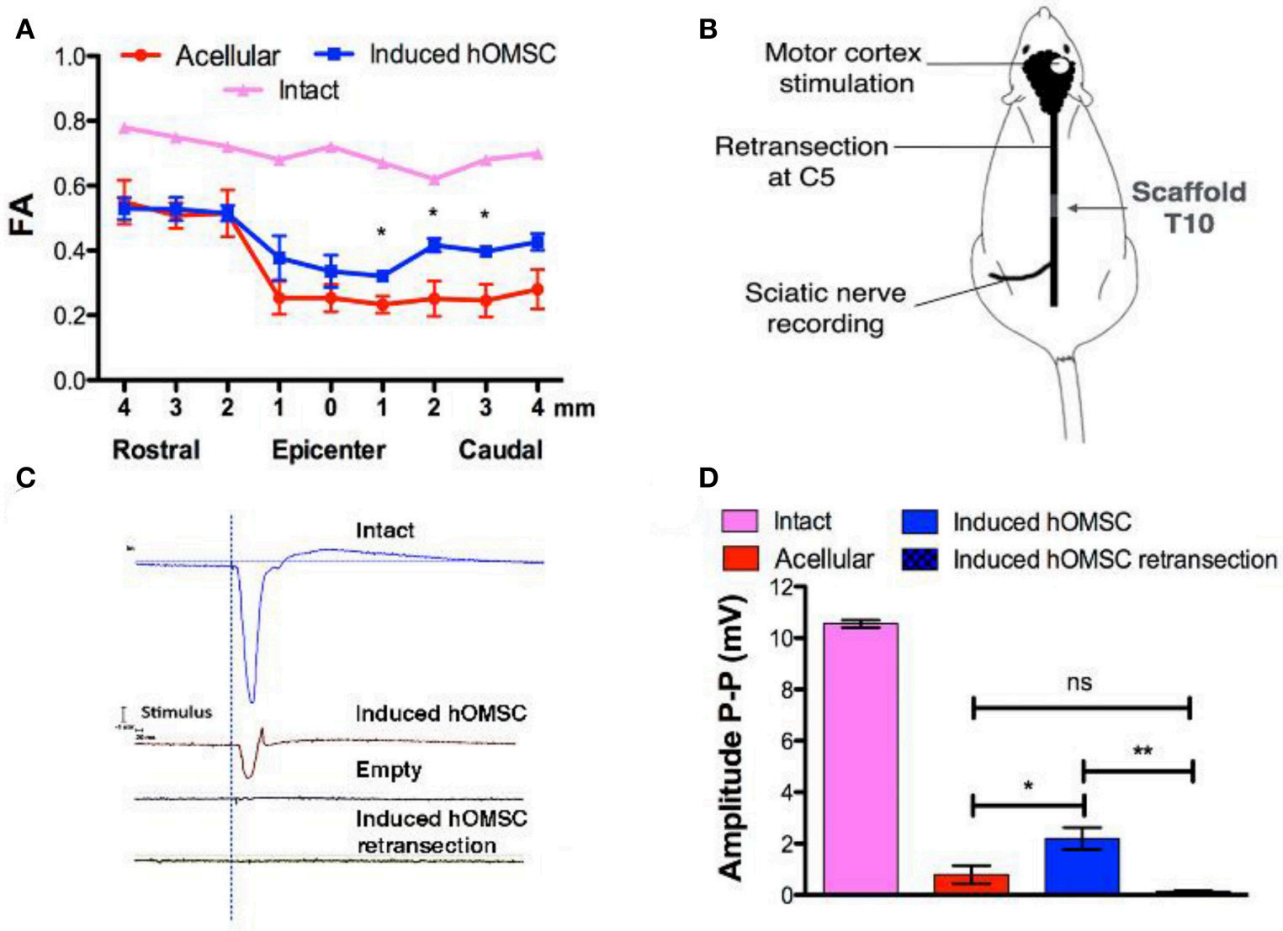
*In vivo* imaging and electrophysiology. **(A)** FA analysis obtained from MRI data of intact rats, rats treated with induced constructs (*n* = 3, mean ± SEM) and rats treated with acellular scaffolds (*n* = 3/group, mean ± SEM). Data are presented as mean ± SEM, one-way ANOVA with Bonferroni *post-hoc* test evaluated statistical differences between the groups at each respective position (^*^p < 0.05). **(B)** Electrophysiology scheme. The rat motor cortex was stimulated by single spikes. The contralateral sciatic nerve was exposed and MEPs were recorded. Following recording, the spinal cord was retransected at C5 and stimulation and recording were performed again to verify signal propagation through the spinal cord. **(C)** Representative recordings of the sciatic nerve in intact rats (blue), rats treated with induced constructs (brown) or with acellular scaffolds (black), and re-transected rats treated with induced constructs (green). **(D)** Quantification of MEP amplitudes in rats treated with induced constructs (*n* = 3), acellular scaffold (*n* = 3) and retransected induced constructs (*n* = 3). Data are presented as mean ± SEM, one-way ANOVA with Bonferroni *post-hoc* test evaluated statistical differences between the groups (^*^p < 0.05, ^**^p < 0.01).

To determine the functional basis of the observed recovery, structural analysis of the spinal cord and electrical signal propagation were evaluated *in vivo*. Temporal structural remodeling of the spinal cord was assessed by MRI diffusion tensor imaging (MRI-DTI) performed 3 and 56 days after surgery in the same animals. In all cases, no fibers connecting the injured spinal cord were observed 3 days after transection with our resolution, suggesting complete transection. Complete transection was also verified using CLARITY (Supplementary Video 5). In contrast, partial connection of rostral and caudal fibers was only present on day 56 in the same rats treated with induced hOMSCs (Supplementary Videos 6, 7). On day 56, fractional anisotropy (FA) recordings 1–4 mm caudal to the injury site were significantly higher in induced-hOMSC construct-treated rats compared to the acellular group, but were still lower than in intact rats (intact rats exhibit FA of 0.7, indicative of organized spinal cord tissue with high anisotropy. Induced hOMSC exhibited FA of 0.32–0.39, higher than the acellular group with FA between 0.23 and 0.25, *p* < 0.05, Figure 3A, Supplementary Figure 4).

To assess functional electrical transmission, motor cortices of rats treated with either induced-hOMSC constructs or acellular scaffolds were stimulated with single pulse stimulation. Motor-evoked potentials (MEPs) were recorded from the isolated sciatic nerve at the hind limb level (Figure 3B). Functional electrical transmission assessments demonstrated high-amplitude signals propagating through the sciatic nerve of rats treated with induced constructs, vs. hardly detectable signal propagation in the acellular group (2.19 ± 0.43 and 0.79 ± 0.35 mV, respectively, a statistically significant difference *P* < 0.04; Figures 3C,D). The electric signal in the former group was abolished by a second transection at C5 rostral to the initial transection (0.14 ± 0.05 mV, statistically significant difference vs. hOMSC measurement before retransection, *P* < 0.009 and non-significant difference vs. the acellular group). This suggests the connectivity between the motor cortex through the spinal cord to the sciatic nerve was exhibited in the induced group and was severed by transection at an arbitrary midpoint.

### Re-establishment of neuronal integrity and glial scar inhibition underlie the observed recovery

**Figure 4:**
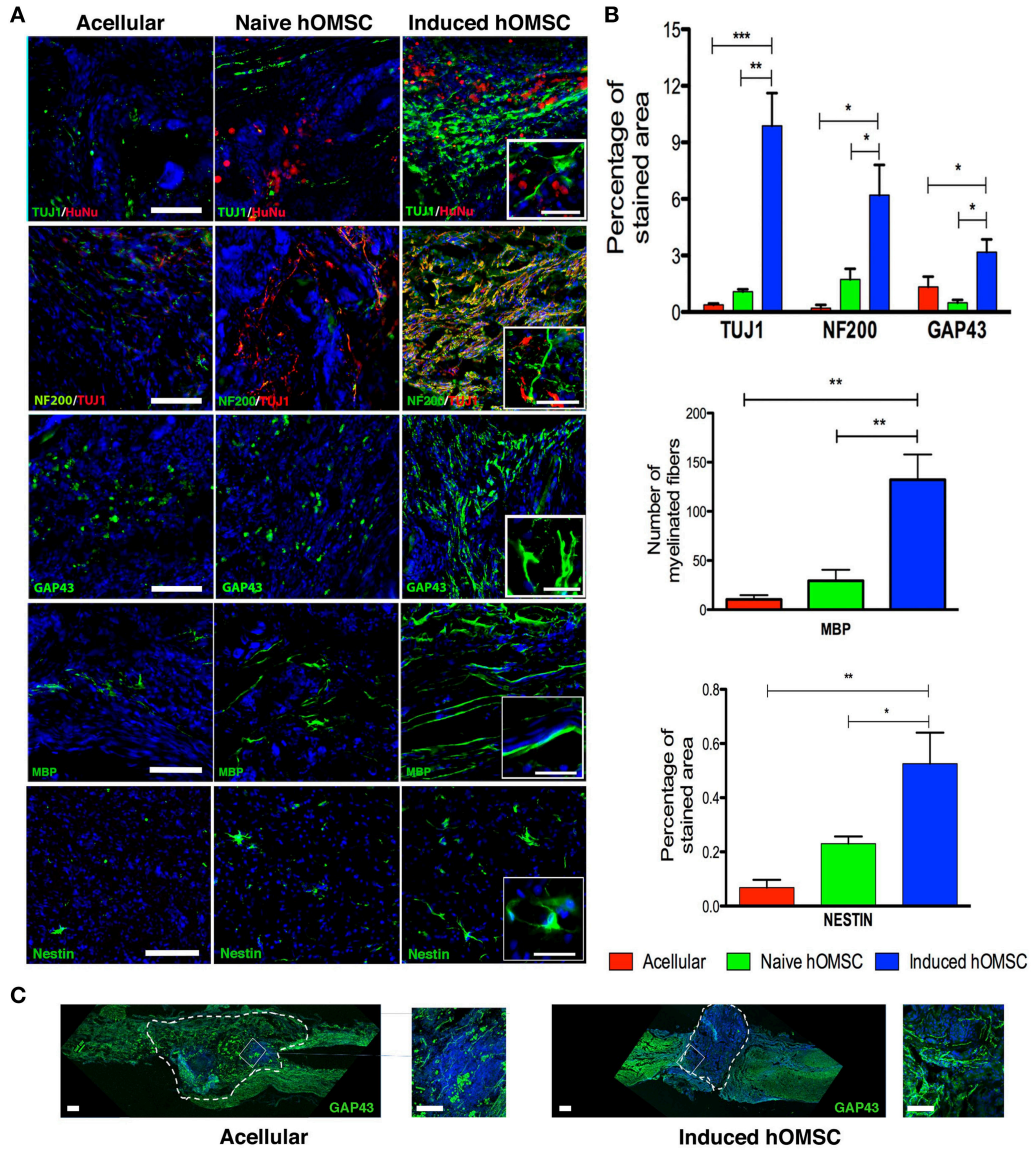
Spinal cord immunofluorescence demonstrating neuronal integrity and axonal elongation-associated markers. DAPI is marked in blue in all immunohistochemistry images. **(A)** Images of spinal cord immunofluorescence staining 8 weeks after implantation of induced constructs and acellular constructs (in descending order): Human nuclear staining, TUJ1 and NF200, GAP43, MBP, nestin and spinal cord beta III tubulin. Scale bar = 200 μm, magnification insets scale bar = 40 μm, *n* = 5/group. **(B)** Computer-based quantification of staining (*n* = 5/group). Top—axonal and neuronal axonal elongation markers, middle—MBP-positive elongated processes, bottom—nestin-positive cells. **(C)** Immunofluorescence staining of longitudinally sectioned spinal cord tissue in rats with acellular PLLA/PLGA scaffolds (left) or induced constructs (right) 8-weeks after lesion. Dotted line indicates scaffold area. Axonal elongation marker GAP43 (green) and DAPI (blue), scale bar = 200 μm, zoom in inset = 50 μm. Data are presented as mean ± SEM, one-way ANOVA with Bonferroni *post-hoc* test evaluated statistical differences between the groups (^*^p < 0.05, ^**^p < 0.01, ^***^p < 0.001).

**Figure 5:**
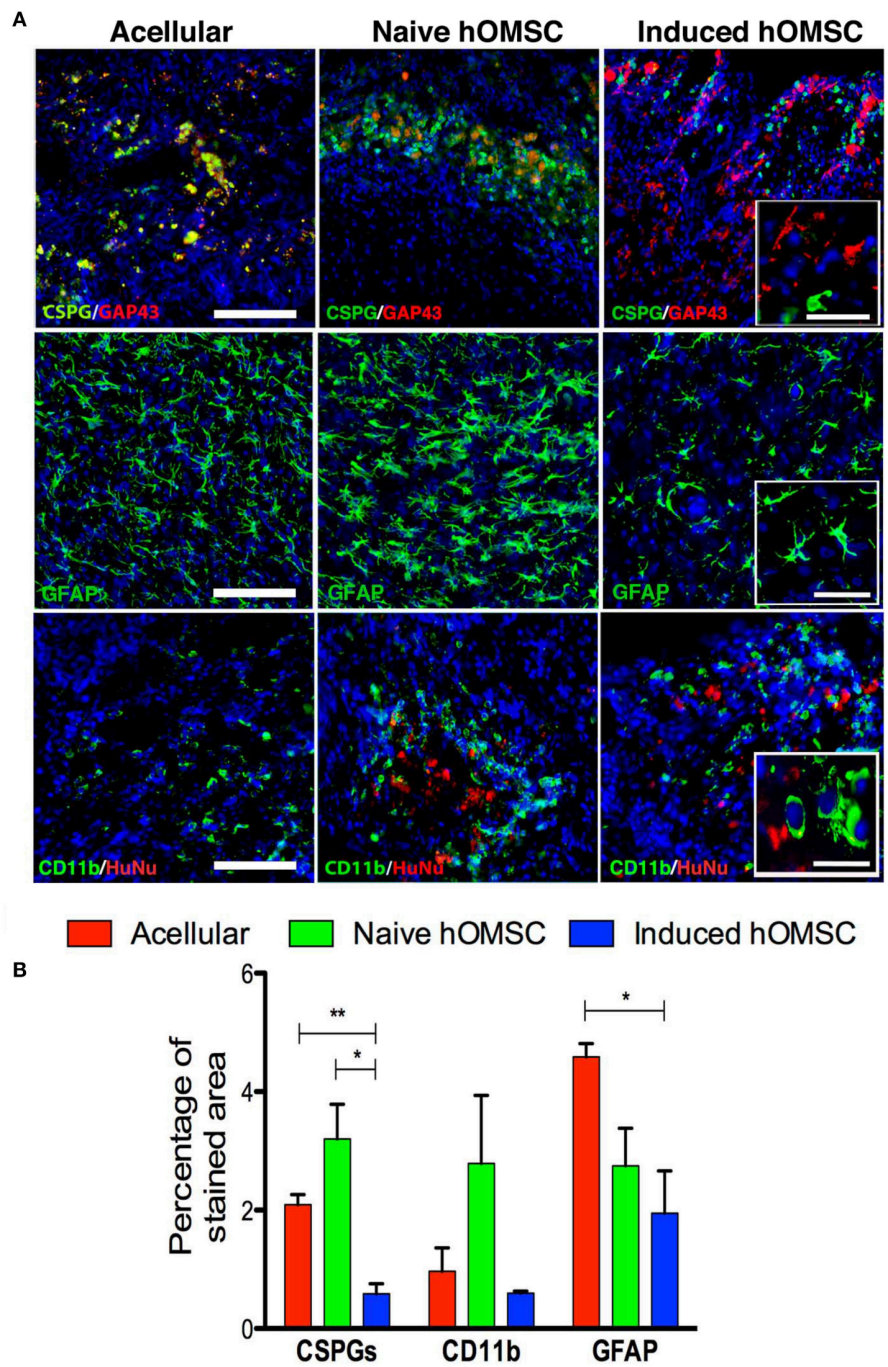
Spinal cord expression of glial scar and inflammation-associated markers. **(A)** Spinal cord immunofluorescence staining 8 weeks after implantation of different treatment groups (in descending order): CSPGs and GAP43, and GFAP and CD11b, DAPI is marked in blue in all immunohistochemistry images (scale bar = 200 μm, magnification insets scale bar = 40 μm, *n* = 5/group). **(B)** Computer-based quantification of staining (*n* = 5/group). Data are presented as mean ± SEM, one-way ANOVA with Bonferroni *post-hoc* test evaluated statistical differences between the groups (^*^p < 0.05, ^**^p < 0.01).

Histology and quali-quantitative immunofluorescence tools were then used to identify cellular mechanisms associated with the recovery of the neuronal circuitry at the injury/implantation site (Figures 4, 5). While some degree of tissue cavitation was observed at the injury site in all the evaluated groups, new tissue with an organized ventral white matter structure was identified in the induced hOMSC construct-treated group only. Neuronal markers beta-III tubulin (TUJ1) and neurofilament-200 (NF200) showed increased expression at the implantation site of the construct bearing induced hOMSCs, but were barely identified in the acellular or naïve groups (Figure 4A). Moreover, 10-fold increase in TUJ1 levels were observed in regions caudal to the lesion site in the induced-construct treated animals when compared to the acellular group (Figure 4A). Similar expression profiles were obtained for the axonal elongation marker NF200, GAP43, myelin basic protein (MBP) and the neuroprogenitor marker nestin (6, 3, 8–15, and 3–6 fold increase respectively to the acellular group; Figures 4A,B). Immunofluorescence staining of longitudinally sectioned spinal cord tissue samples from rats treated with an acellular PLLA/PLGA scaffold or induced construct showed GAP43-positive fibers passing through the dense glial scar tissue into the PLLA/PLGA scaffold. More remarkable GAP43-positive fibers were present in higher numbers in the induced construct group as compared to the acellular group (Figure 4C).

The lowest levels of the glial scar marker chondroitin sulfate proteoglycan (CSPG) as well as GFAP and were measured in the induced hOMSC group as compared to all other groups by 3- to 5-fold reduction (Figure 5A). GFAP levels and the number of CD11b-positive microglial cells were also reduced in this group, while marked microglial presence was noted around naïve hOMSCs (Figure 5A). CD11b was also markedly reduced but to no statistically significant degree (Figure 5B). To evaluate the migration of induced-hOMSC into the spinal cords, constructs engineered with GFP-labeled cells were implanted and monitored. While a few GFP-positive cells were identified at a distance of up to 4 mm both rostral and caudal to the implantation site, 28 days after transplantation, the majority of cells remained within the implanted construct area (Supplementary Figure 5).

## Discussion

In this study, we report that transplantation of artificial tissue constructs secreting trophic factors formed a growth-permissive microenvironment that counteracted inhibitory signals and promoted connectivity restoration across the injury spinal cord site (Jones et al., 2001). In most cases, these effects translated to substantial functional improvement, enabling paraplegic rats to walk independently and regain sensory perception (Figures 2,3). Functional recovery (BBB scores between 14 and 21) was achieved in 0, 10, 40, and 42% of the rats implanted with no construct or with an acellular, naïve and induced construct, respectively (Figures 2C,D). Specifically, 5 of 12 rats (42%) treated with the induced constructs demonstrated BBB scores exceeding 17, a compiled reflection of improved coordinated gait, plantar placement, weight support, recovery of toe clearance, trunk stability, and predominant parallel paw and tail position, suggesting regained cortical motor control (Basso et al., 1995; Li et al., 2014). Previous attempts to treat SCI have thoroughly evaluated a large variety of cells, both in combination with TE and with ectopic expression of therapeutic genes (Tetzlaff et al., 2011; Granger et al., 2014). Here we engineered a multi-effector construct exhibiting an average BBB score of 9.7, with some animals exhibiting 19–20 BBB scores and normal motor coordination (Figure 2). Yet, focus on the mean BBB scores does not reflect the binary or on/off outcome of our treatment. The induced constructs promoted remarkable recovery in 42% of the rats, and show no efficacy in the remainder of the rats within the same group. This binary effect compels further investigation, since understanding of the underlying mechanisms causing substantial improvement in some animals and no practical improvement in others can render this method into an effective treatment. In contrast, transected but untreated rats from the none group showed limited spontaneous recovery, confirming again the complete transection of the spinal cord in the tested groups. Interestingly, although most of the rats transplanted with empty scaffolds did not show major improvements (80% with BBB <7), 20% of the rats treated with scaffold only exhibited substantial improvements, but to a lesser degree than the other groups. Indeed, previous studies demonstrated that scaffolds alone are able to promote axonal elongation and functional recovery in rodents (Lavik et al., 2002; Teng et al., 2002) and primates (Slotkin et al., 2017).

The recovery reported here for the induced group was also accompanied by an improvement in sensory responses to gross external stimuli in 75% of the treated rats, while all the other groups failed to show such responses (Figure 2F). Furthermore, our findings (Figures 2,3) resonate with 11 studies reporting a positive correlation between electrical conduction and functional effects elicited by cell transplants after SCI (Granger et al., 2014). MEPs travel in both dorsolateral and ventral pathways and are routinely used to evaluate reticulospinal and corticospinal tract integrity (Garcia-Alias et al., 2006). The amplitudes of the propagated MEPs recorded in the induced group were lower than those observed in the intact animal, but significantly higher than those of the acellular group. This suggests at least partial connectivity between the motor cortex and motor neurons innervating the hindlimbs Moreover, MEP propagation was completely abolished after rostral spinal cord re-transection, indicating the motor evoked potential traveled through corticospinal tracts across the lesion, and rejecting possible assumptions correlating functional recovery with spinal locomotion effects (Fehlings et al., 1988; Courtine et al., 2009; Ziegler et al., 2011; Lu et al., 2012).

Histological, CLARITY and MRI-DTI analyses provided additional evidence of the complete interruption of axonal bundles at the transected level in non-walking animals, while fibers bridging the injury site were detected in walking rats (Figures 3,4, Supplementary Figure 3). The clearly abnormal stepping pattern typically observed in the induced group animals may suggest lack of restoration of propriospinal or modulatory control, or a hypersensitivity phenomenon. Yet, partial structural restoration of the connectivity, combined with small-amplitude MEPs were sufficient to elicit substantial functional recovery in the whole animal (Figure 3). The explanation to this dissimilarity between this substantial functional recovery and the limited recovery of the electro-physiological properties (as well as the axonal connectivity as provided in the MRI study) is still unknown. It might be attributed to partial reconnection to a spinal cord pattern generator that governs the locomotion (Rybak et al., 2006).

MRI-FA values demonstrated improved directionality of axon tracts in the induced-hOMSC group, which positively correlated with TUJ1, NF200, GAP43, and MBP staining and motor function, indicating active axonal elongation processes as previously reported (Figures 2–4, 3A, Supplementary Figure 4; Nevo et al., 2001; Kozlowski et al., 2008; Kelley et al., 2014). At the same time, the induced hOMSC group displayed the lowest levels of the axonal elongation inhibitory factor, CSGPs, and the lowest degree of co-localization with GAP43 (Figure 4). In contrast, strong co-localization of CSPGs and GAP43 was observed in all the other control groups, suggesting axon growth neutralization or collapse, highly resembling those of the dystrophic end bulbs or “sterile clubs” first reported by Ramon y Cajal (Ramón Y Cajal, 1928; Davies et al., 1999). In rats treated with the acellular PLLA/PLGA scaffolds and induced constructs, GAP43-positive fibers traversed the dense glial scar tissue into the scaffold area, suggesting that the scaffold itself plays an important role in tissue regeneration, resonating with the findings by Teng et al. who reported that the scaffold alone promoted functional recovery by reducing epidural and glial scar formation (Teng et al., 2002). Yet, as compared to the acellular group, a larger number of GAP43-positive fibers were detected in the induced construct group, probably due to paracrine activity of the induced hOMSCs within the scaffold (Figure 4C). In line with these observations, astrocytes, and to some extent, microglial reactivity were reduced, potentially avoiding a hostile environment for neuronal plasticity and repair (Figure 5). In addition, induced constructs may support recruitment and/or proliferation of nestin-positive progenitors (Figure 4), and their respective differentiation at the injury site (Matsumura et al., 2010). Grafted cells were mostly detected at implantation site, indicating the effectiveness of the scaffolds in retaining the cells and enhancing the local paracrine effect mediated by secreted factors.

Analysis of the induced constructs by a protein array provided additional evidence of a paracrine effect, with increased secretion of BDNF, NT-3/4, GDNF, VEGF, HGF, SDF-1, and G-CSF (Kitamura et al., 2011; Jeong et al., 2012; Silva et al., 2014). BDNF, NT-3/4, VEGF, and HGF have been shown to induce the growth of neurites and NF200/GAP-43 axons, and to promote myelination and regeneration of CST, rubrospinal, and reticulospinal tracts. These factors were also associated with reduced inflammation, glial reactivity, and reduced expression of the glial scar molecules in the spinal cord (Kobayashi et al., 1997; Liu et al., 1999; Facchiano et al., 2002; Jin et al., 2002; Tuszynski et al., 2003; Kitamura et al., 2011; Jaerve et al., 2012; Jeong et al., 2012; Kadoya et al., 2016). SDF-1 can promote axon outgrowth in the presence of myelin inhibitors, as well as support and attract endogenous nestin-positive neural precursor cells to the injury site (Jaerve et al., 2012), as observed in our analysis.

The clear binary or on/off effect observed within the treated animals, ranging from unresponsive rats to highly responsive ones may have been the results of heterogeneous or variable 3D cell seeding and differentiation within the scaffold. More importantly, their precise implantation position at the spinal level, may have selectively induced partial repair and reconnection of specific spinal tracts, as previously described (Kobayashi et al., 1997; Liu et al., 1999; Facchiano et al., 2002; Jin et al., 2002; Tuszynski et al., 2003; Kitamura et al., 2011; Jaerve et al., 2012; Jeong et al., 2012). Since the minimum requirements for eliciting substantial recovery have yet to be defined, subtle differences in the scaffold and its position related to the spinal cord stumps may have selectively favored restoration of some but not all tracts.

Considering the complexity of tissue repair in spinal cord injuries, it is likely that the observed functional changes were elicited by the synergistic activity of several factors differentially secreted by induced constructs leading to neuroprotection, gliomodulation, and consequential stimulation and/or repressed inhibition of axonal elongation processes (Tetzlaff et al., 2011). Furthermore, a minor recovery of the electro-physiological properties probably induced by axonal elongation or by neuroprotective mechanisms elicited functional recovery, even in the scaffold only group. As demonstrated in this study, immediate provision of these cues after injury minimizes secondary degeneration and stimulates endogenous regenerative/repair mechanisms. The treatment design exploited an implant fabricated of FDA-approved, biocompatible, and biodegradable materials with easily accessible adult stem cells, which are induced through a medium-based protocol. This multi-effector approach showed encouraging preliminary results and warrants further investigation to shed light on the mechanisms underlying the observed recovery, to enable improved efficacy and to define the intervention optimal for treatment of spinal cord injury.

## Ethics statement

Cells obtained from human source: This study was carried out in accordance with the recommendations of the Institutional Helsinki Committee at the Baruch Padeh Medical Center, Poria, Israel with written informed consent from all subjects. All subjects gave written informed consent in accordance with the Declaration of Helsinki. The protocol was approved by the Institutional Helsinki Committee at the Baruch Padeh Medical Center, Poria, Israel. Animal procedures were carried out in accordance with the protocol approved by Technion IACUC. MRI studied were done in accordance with protocol approved by Tel-Aviv University IACUC.

## Author contributions

JG, ES, SG, IM, SP, DO, and SL: Designed the experiments. JG, ES, AS, and SG: Performed the experiments. IA: Contributed reagents. JG, ES, SG, SP, DO, and SL: Wrote and edited the paper.

### Conflict of interest statement

The authors declare that the research was conducted in the absence of any commercial or financial relationships that could be construed as a potential conflict of interest.
