# Supplementary material for: Implantation of 3D Constructs Embedded with Oral Mucosa-Derived Cells Induces Functional Recovery in Rats with Complete Spinal Cord Transection
Source: Front Neurosci. 2017 Oct 31;11:589. doi: 10.3389/fnins.2017.00589 (PMC5671470; doi:10.3389/fnins.2017.00589)
Supplement: Supplementary file 8 [file DataSheet1.docx]

**Supplementary information:**

**Supplementary table T1 – Proteomic array analysis showing expression fold-change of 80 secreted proteins in induced vs. naïve constructs**


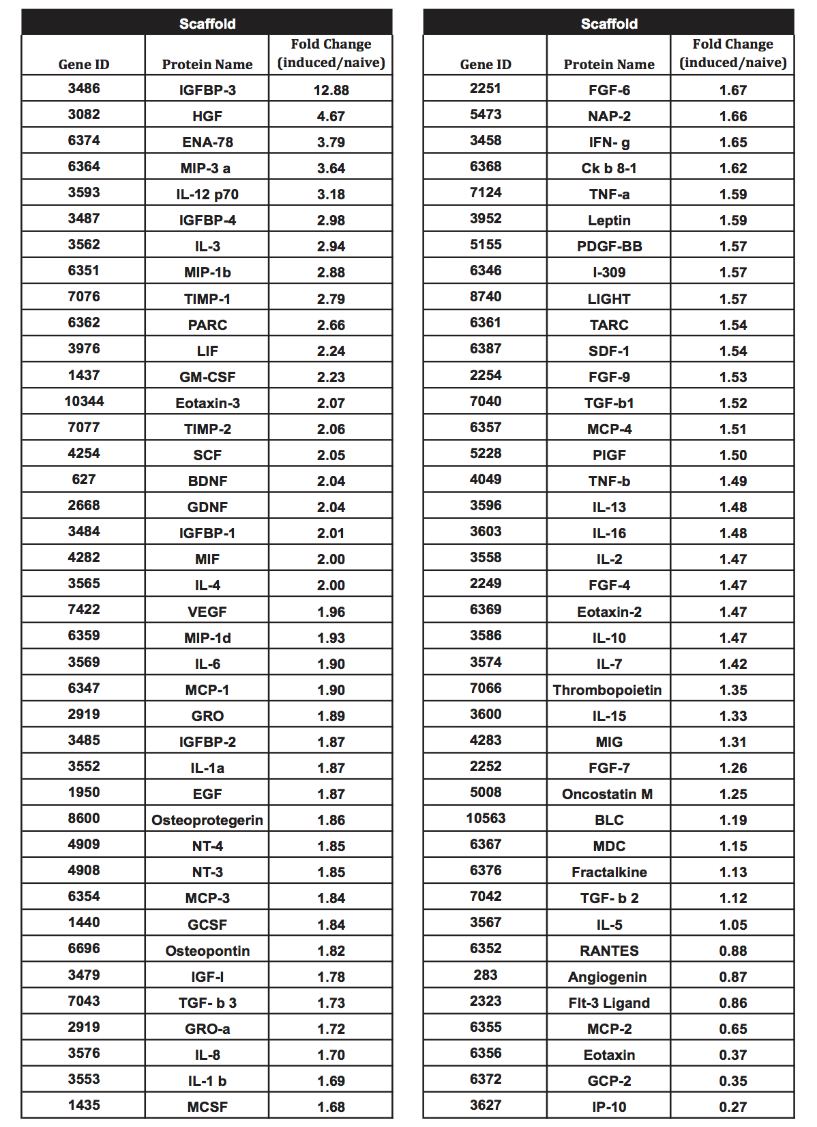


**Supplementary table T2 – Animals participating in study**

|  | Initial #of rats | Premature death (during first 4d) | BBB>4 at first measurement | Survived more than 5 days | % included (Survival wk1) | %Survival after 5 wks |
| --- | --- | --- | --- | --- | --- | --- |
| None | 17 | 1 |  | 16 | 94% | 53% |
| acellular | 14 | 4 |  | 10 | 71% | 57% |
| naïve | 7 | 2 |  | 5 | 71% | 71% |
| induced | 15 | 1 | 2 | 12 | 80% | 73% |

Excluded participants include animals with BBB>4 at first measurement and premature death (survived 5 days or less). All other animals participated in the experiment. Early termination of animals was a result of self-mutilation or weight loss.

**
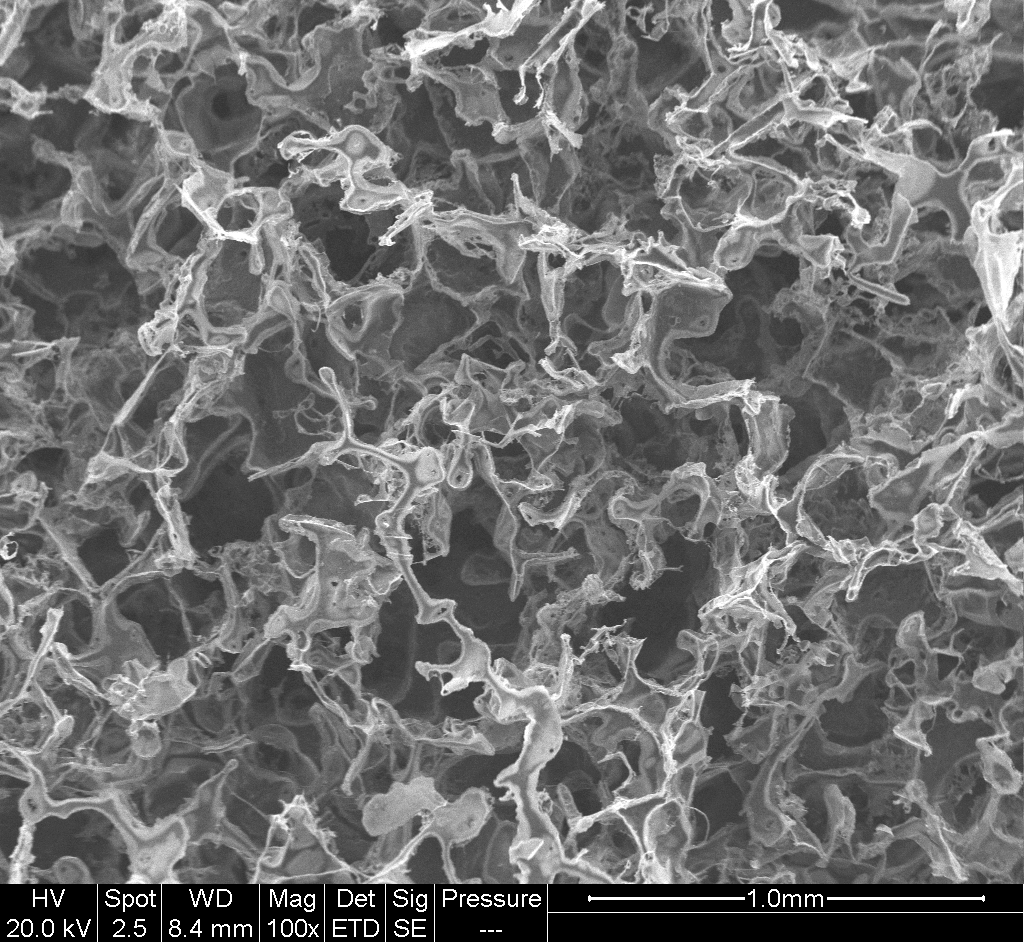
**

**Supplementary figure S1 – PLLA/PLGA scaffold micro-structure.** Micro-pores in scaffold allow cells adhesion and nutrient delivery. The construct is stiff enough to enable implantation.


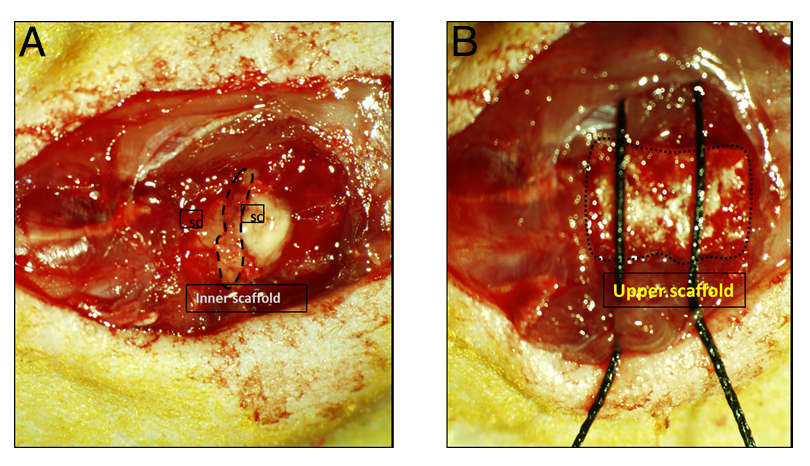


**Supplementary figure S2 – Construct implantation procedure.** The construct is shown implanted between the two transected spinal cord stumps (A). The sealing PLLA/PLGA scaffold is placed over the transection area and sutured in place (B)


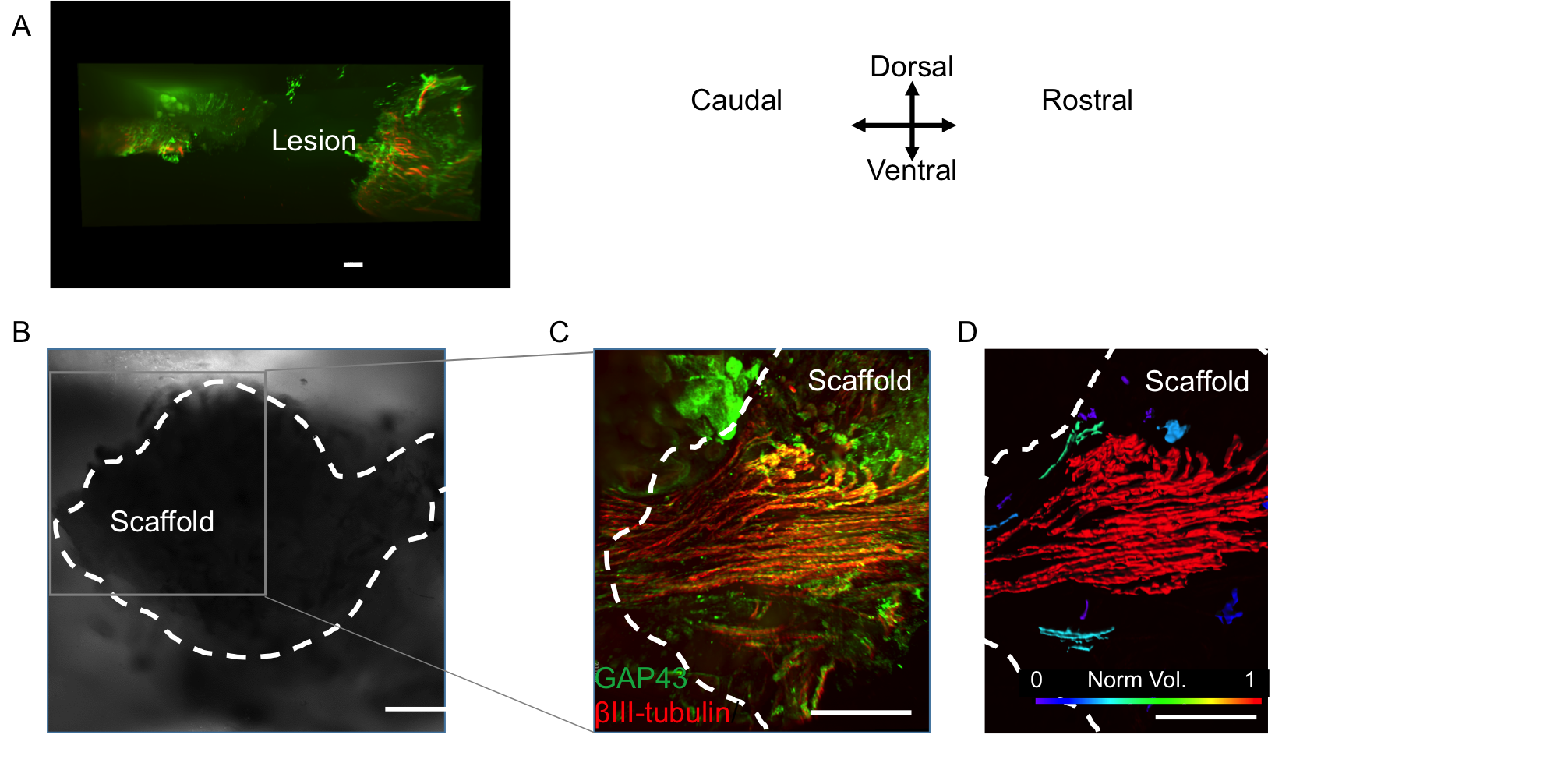


**Supplementary Figure S3 – CLARITY analysis of spinal cords with and without constructs.** (A) – CLARITY image of transected spinal cord without scaffold showing a gap in the transection area. (B) – Brightfield CLARITY image of a scaffold implanted in the transection area. (C) – staining of axons in the scaffold penetrating into the gap. βIII-tubulin (red) and GAP43 (green) staining shows elongated neuronal fibers penetrating into the scaffold area. (D) – Image processing of immunostaining: automated segmentation of elongated elements penetrating into the scaffold area (connected overall volume is indicated by color, red indicates maximum volume of elongated fibers elements ). Scale bar – 200um.

**
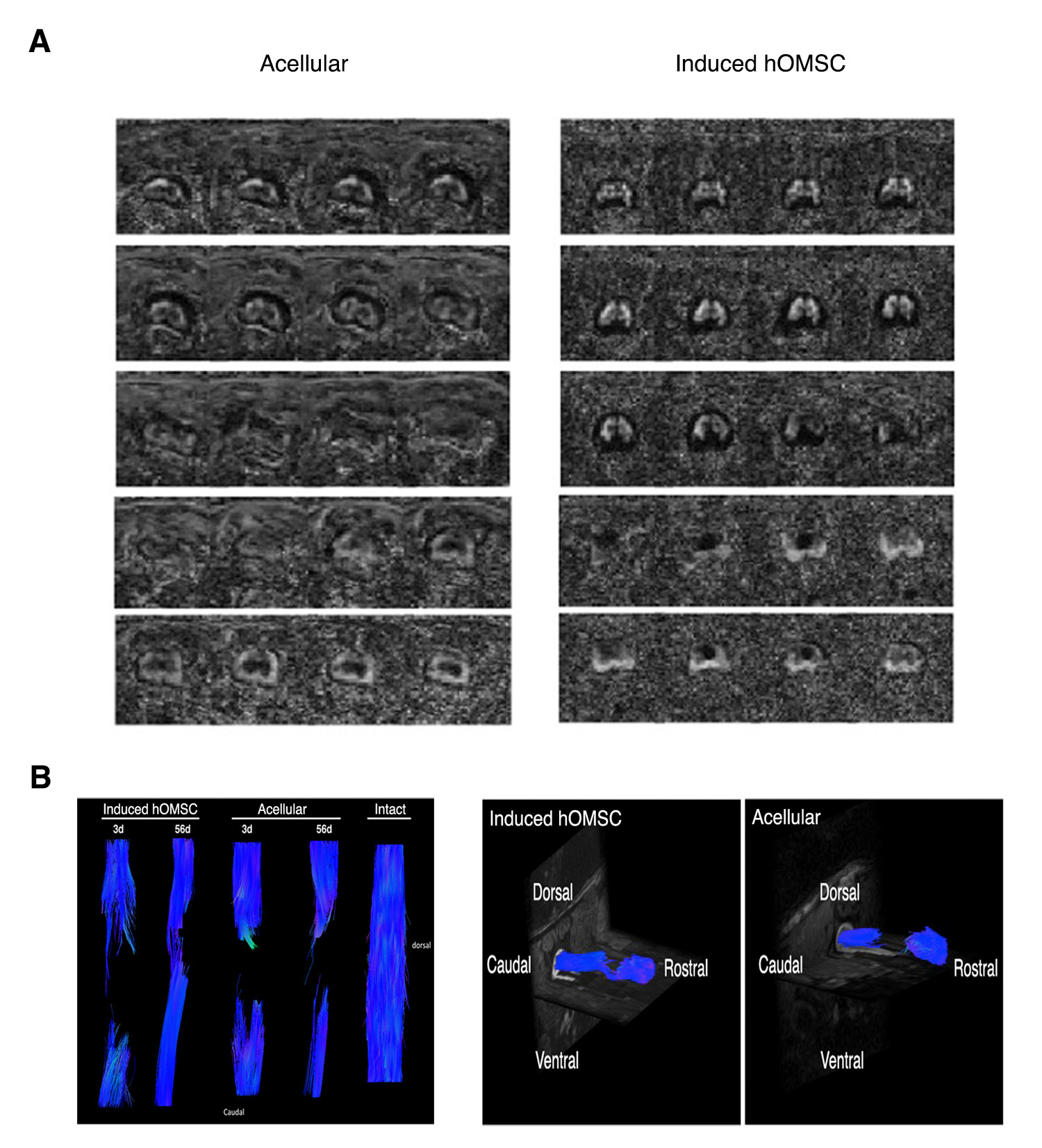
**

**Supplementary Figure S4 – (A) Fractional anisotropy maps.** Rostro-caudal serial sections obtained by MRI scans of rats spinal cords treated with an induced-construct (right) or acellular scaffold (left). Lighter colors represent higher FA values indicating the presence of soft tissue with distinct rostral-caudal directionality. (B) MRI-DTI fiber tracking on days 3 and 56, in rats treated with induced constructs or acellular scaffold showing fibers traced in the rostral-caudal direction. No connectivity was observed 3 days after surgery in both the acellular and induced construct groups. Induced group exhibited partial connectivity after 56 days. intact rats served as a reference. Top – rostral. (left panel). 3D rendering of fibers overlaid on day 56-anatomical MRI data for rats treated with induced constructs versus rats treated with acellular scaffold (right panel).


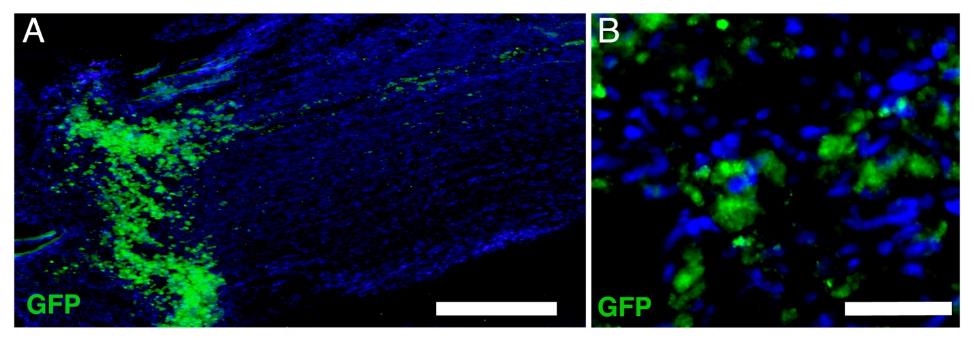


**Supplementary Figure S5 -** Immunofluorescence image and zoom in caption of GFP-labeled cells within the induced-construct, as observed at the end of the experiment (A-B). DAPI is marked in blue in all immunohistochemistry images. (Day 56).

**Supplementary video 1 –** Video of confocal microscopy z-stack, from bottom up, of induced GFP-labeled hOMSCs on a PLLA/PLGA scaffold.

**Supplementary video 2 –** Video showing motor function of representative rats: Transection only after one day and 43 days, Acellular construct after 3 days and 43 days, and induced construct after 3 days and 43 days.

**Supplementary video 3 –** Video showing reproducibility of results obtained with the induced construct. Four rats implanted with induced hOMSCs constructs exhibited similar motor functions.

**Supplementary video 4 –** Video showing reproducibility of results obtained with acellular scaffolds. Four rats implanted with acellular constructs exhibited similar motor functions.

**Supplementary video 5 –** 3D rendering of spinal transection following CLARITY application

**Supplementary video 6 -** 3D rendering of fibers overlaid on day 56-anatomical MRI data for rats treated with induced constructs.

**Supplementary video 7 -** 3D rendering of fibers overlaid on day 56-anatomical MRI data for rats treated with acellular constructs.
